# Saliva Is a Sensitive and Accessible Sample Both for SARS-CoV-2 Detection and for the Evaluation of Treatment Effectiveness in Follow-Up Studies

**DOI:** 10.3390/v16071040

**Published:** 2024-06-27

**Authors:** Eleonora Lalle, Valentina Mazzotta, Giuseppe Sberna, Lavinia Fabeni, Anna Rosa Garbuglia, Ilaria Mastrorosa, Alessandra D’Abramo, Emanuele Nicastri, Enrico Girardi, Andrea Antinori, Fabrizio Maggi, Licia Bordi

**Affiliations:** 1 Laboratory of Virology and Biosafety Laboratories, National Institute for Infectious Diseases Lazzaro Spallanzani—IRCCS, 00149 Rome, Italy; 2 Clinical and Research Department, National Institute for Infectious Diseases Lazzaro Spallanzani—IRCCS, 00149 Rome, Italyilaria.mastrorosa@inmi.it (I.M.);; 3 Scientific Direction, National Institute for Infectious Diseases Lazzaro Spallanzani—IRCCS, 00149 Rome, Italy

**Keywords:** SARS-CoV-2, molecular assay, saliva, COVID-19 diagnosis, monoclonal antibodies, antiviral therapy, follow-up studies

## Abstract

Despite emerging evidence indicating that molecular SARS-CoV-2 tests performed on saliva have diagnostic sensitivity and specificity comparable to those observed with nasopharyngeal swabs (NPSs), most in vivo follow-up studies on the efficacy of drugs against SARS-CoV-2 have been performed on NPSs, not considering saliva as a possible alternative matrix. For this reason, in this study, we used, in parallel, saliva and NPS samples for the detection of SARS-CoV-2 by real-time RT-PCR in patients receiving Tixagevimab/Cilgavimab, Nirmatrelvir/Ritonavir, or Sotrovimab as a treatment against SARS-CoV-2. Our results showed a good correlation between the NPS and saliva samples for each drug; moreover, comparable changes in the cycle threshold (Ct) levels in saliva and NPSs were observed both 7 days and 30 days after treatment, thus confirming that the saliva represents a good matrix for in vivo follow-up studies verifying the effectiveness of treatments against SARS-CoV-2.

## 1. Introduction

Despite its prominence in early scientific records, the usefulness of saliva as a respiratory specimen has been de-emphasized over the past century. Before the COVID-19 pandemic, several studies had already refreshed the potential of saliva for detecting seasonal respiratory viruses. Notably, despite the positive results obtained by these studies, little work has been carried out to further elucidate the possible role of this matrix for diagnostic purposes [1]. Increasing evidence has emerged during the COVID-19 pandemic indicating that molecular tests performed on saliva have diagnostic sensitivity and specificity comparable, or even superior, to those observed with a nasopharyngeal swab (NPS) for SARS-CoV-2 detection [2,3,4,5,6,7,8,9]. Based on this evidence, numerous efforts have been made to develop and implement saliva-based diagnostic tests aimed at expanding the use of saliva in clinical settings [10].

Looking towards the future, as saliva uniquely contains both respiratory secretions and immunological components, it has potentially wide applications, ranging from clinical diagnostics to post-vaccine disease burden and immunity surveillance. Due to its easy, painless self-collection and viral stability, saliva represents a suitable alternative sample, especially in community mass-screening programs and the longitudinal sampling of hospitalized individuals aimed at monitoring viral load dynamics and treatment response.

Since the beginning of the SARS-CoV-2 pandemic, the entire scientific world has been developing vaccines, representing an optimal method of pre-exposure prophylaxis. Nevertheless, after four doses, immunocompromised individuals show a reduced vaccine response [11,12,13,14]. In this context, regimens of monoclonal antibodies (mAbs) or antiviral agents have been developed, which are useful as a treatment against COVID-19 and provide passive immunization to boost immunity against SARS-CoV-2.

Over time, several drugs have been used for the treatment of SARS-CoV-2, including the direct-acting antivirals (DAAs) remdesivir (RDV), molnupiravir (MNP), and ritonavir-boosted nirmatrelvir (NRM), as well as anti-spike monoclonal antibodies (mAbs; i.e., Tixagevimab/Cilgavimab or Sotrovimab) [15].

Numerous studies have analyzed how the efficacy of mAbs and antivirals, initially produced against early SARS-CoV-2 variants, has changed over time, with new variants, especially the Omicron subvariants [16,17,18,19,20]. Scientific evidence highlighted that continued virus evolution has progressively decreased or abolished mAbs’ neutralization activity, with few cases of restored activity against the currently dominating virus lineages [21]; on the other hand, DAA seemed to have maintained their activity across multiple variants due to the high conservation of the targeted viral enzymes [22].

In most of these in vivo follow-up studies, the virological outcome of treatment with mAbs, antivirals, or multiple combination therapies has been evaluated by measuring viral loads in NPSs [23,24,25,26,27].

Here, we investigated the possible use of saliva samples as a surrogate of NPSs in in vivo follow-up studies in COVID-19 patients receiving Tixagevimab/Cilgavimab, Nirmatrelvir/Ritonavir, or Sotrovimab, in order to establish the effectiveness of different treatments.

## 2. Materials and Methods

### 2.1. Study Population

Sixty patients who accessed the National Institute for Infectious Diseases “L. Spallanzani” from March to May 2022 were included in this study, with a median time from symptom onset of 3 days (IQR 2–4). None of the patients was hospitalized, since all of them had mild-to-moderate COVID-19. The enrolled patients met the Italian Pharmaceutical Agency (AIFA) criteria for eligibility for treatment with monoclonal antibodies or antiviral agents. Treatment allocation was dependent on drug availability, time since symptom onset, and the presence of comorbidities as defined by the AIFA criteria. The patients were selected from a larger cohort previously described [28] based on the administered therapy, homogeneous sex, and a similar mean age: 20 patients received Tixagevimab/Cilgavimab (10 men and 10 female patients, mean ± SD: 58.80 ± 15.83), 20 Nirmatrelvir/Ritonavir (10 men and 10 female patients, mean ± SD: 66.80 ± 12.52), and 20 Sotrovimab (10 men and 10 female patients, mean ± SD: 62.95 ± 13.17).

The patients were attested in a follow-up at three time points: before drug administration (T0), on day 7 (T7), and on day 30 (T30) after treatment initiation. Data from the biological samples were used only after complete anonymization.

### 2.2. Sample Collection

Two mL of saliva was self-collected, under medical personal supervision, by passive drooling, spontaneously produced without external stimuli, in sterile containers without any buffer added, at least 30 min after drinking, eating, or brushing one’s teeth. NPSs were put into sterile tubes containing 2–3 mL of viral transport media (Copan UTM^®^ Universal Transport Medium, Copan Diagnostics Italia s.p.a., Brescia, Italy). The NPS and saliva samples were collected simultaneously and immediately processed.

### 2.3. SARS-CoV-2 Real-Time PCR

The NPS and saliva samples were tested using an Alinity mSARS-CoV-2 AMP assay (Abbott Diagnostics GmbH, Wiesbaden, Germany), targeting RdRp and N. Briefly, the NPSs were directly loaded on the Alinity instrument using a lysis solution, while the saliva samples had been previously diluted with the Alinity m Specimen Diluent (diluent volume ratio of 1:1.25). 

### 2.4. Sequence Analysis

The genetical characterization of SARS-CoV-2 variants was performed on the saliva and/or NPS samples by Sanger sequencing. The extraction of viral RNA was performed using the automated QiaSymphony system (Qiagen Instruments AG Switzerland, Hilden, Germany), according to the manufacturer’s instructions. The amplified products of the fragment corresponding to the aminoacidic coverage 399–616 of the SARS-CoV-2 spike gene obtained by means of a one-step RT-PCR kit (Qiagen) were sequenced using the Big Dye Terminator Cycle Sequencing kit v3.1 (Applied Biosystems, Foster City, CA, USA) and an automatic DNA sequencer (ABI model 3130 and/or 3500 XL, Applied Biosystems, Foster City, CA, USA). The sequences were compared by alignment with the original Wuhan virus sequence (accession number: NC_045512.2) [7].

### 2.5. Statistical Analysis 

Data management and analyses were performed using GraphPad Prism version 9.3.1 (GraphPad Software, La Jolla, CA, USA). The characterization of the patients enrolled in this study was performed by descriptive analysis.

The correlation between the cycle threshold (Ct) values obtained in the NPSs and the Ct obtained in the saliva was calculated by a linear regression analysis, with negative samples assigned an arbitrary value of 42 Ct, with 40 Ct set as the detection limit of the assay.

## 3. Results

**Figure 1:**
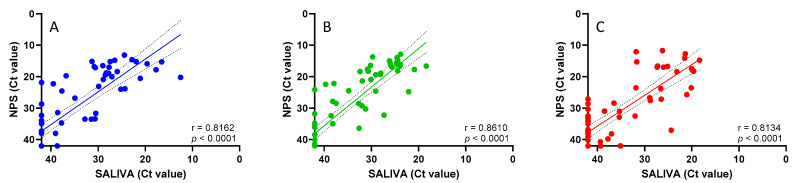
Linear regression analysis in NPS and saliva samples in patients receiving Tixagevimab/Cilgavimab (**A**), Sotrovimab (**B**), or Nirmatrelvir/Ritonavir (**C**).

The study population, genetically characterized as SARS-CoV-2 BA.1 (83.3%), BA.2 (15%), and BA.5 (1.7%) variants, was stratified based on the different SARS-CoV-2 treatments received (20 patients treated with Tixagevimab/Cilgavimab, 20 patients with Sotrovimab, and 20 patients with Nirmatrelvir/Ritonavir), and the correlation between the Ct values obtained in the NPS versus saliva matrices was evaluated (Figure 1). As shown in Figure 1, a statistically significant correlation was observed for both the mAb (Tixagevimab/Cilgavimab: r = 0.8162, *p* < 0.001 panel A; Sotrovimab: r = 0.8610, *p* < 0.001, panel B) and antiviral treatments (Nirmatrelvir/Ritonavir: r = 0.8134, *p* < 0.001, panel C). 

**Figure 2:**
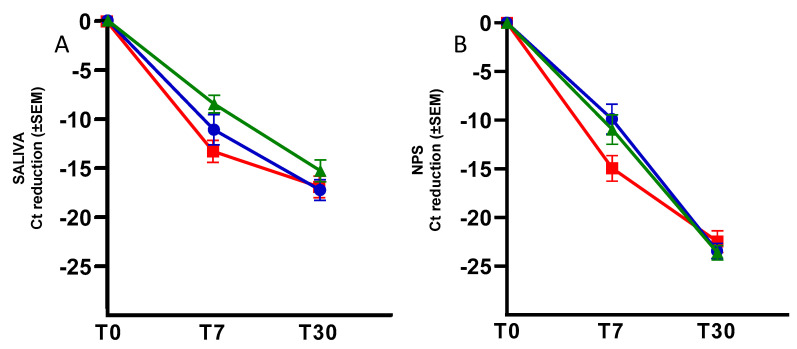
Mean of Ct reduction in saliva samples (**A**) and NPSs (**B**) at T0, T7, and T30, compared to T0, according to the three different treatments. SEM: standard error of the mean. Tixagevimab/Cilgavimab in blue, Sotrovimab in green, and Nirmatrelvir/Ritonavir in red.

Moreover, the Ct reduction during the follow-up period was examined, measuring T7 or T30 minus T0, both in the saliva (Figure 2, Panel A) and NPSs (Figure 2, Panel B).

Specifically, when analyzing the Ct values at T7 minus T0, in the saliva matrix, the mean reduction was −13.23 (SEM: ± 1.13) Ct after treatment with Nirmatrelvir/Ritonavir, −11.12 (SEM: ± 1.54) Ct following Tixagevimab/Cilgavimab, and −8.52 (SEM: ± 0.90) Ct following Sotrovimab. The same analysis was performed on the NPSs, showing a mean Ct reduction of −14.92 (SEM: ± 1.31), −9.89 (SEM: ± 1.53), and −10.96 (SEM: ± 1.51), respectively.

When analyzing the Ct values at T30 minus T0, the saliva matrix showed a mean Ct reduction of −16.86 (SEM: ± 1.10) Ct after treatment with Nirmatrelvir/Ritonavir, −17.27 (SEM: ± 1.04) Ct with Tixagevimab/Cilgavimab, and −15.32 (SEM: ± 1.10) Ct with Sotrovimab. Looking at the NPSs, means of −22.45 (SEM: ± 1.11), −23.44 (SEM: ± 0.77), and −23.62 (SEM: ± 0.74) were observed, respectively.

## 4. Discussion

During the COVID-19 pandemic, a growing consensus emerged on the use of saliva as an effective, stable, low-cost, and non-invasive matrix to detect SARS-CoV-2 RNA [8]. Even though saliva tests were thought to be less sensitive than NPS tests, many studies have since shown that the molecular tests for SARS-CoV-2 detection performed on saliva are comparable in terms of diagnostic sensitivity and specificity to NPSs [3,28,29]. Nevertheless, most in vivo follow-up studies on the efficacy of drugs against COVID-19 have been performed on NPSs, not considering saliva as a possible alternative matrix.

In this study, we analyzed 60 patients receiving Tixagevimab/Cilgavimab, Nirmatrelvir/Ritonavir, or Sotrovimab as an early treatment against COVID-19, testing, in parallel, oral saliva and NPS samples to evaluate the virological outcome of the treatment by real-time RT-PCR.

When stratifying the study population based on the different COVID-19 treatments received, a statistically significant correlation between the saliva and NPS samples was observed for both the mAb (Tixagevimab/Cilgavimab, Sotrovimab) and antiviral treatments (Nirmatrelvir/Ritonavir), confirming that both matrices are eligible for SARS-CoV-2 RNA detection.

Moreover, the mean Ct reduction at T7 minus T0 was comparable in both matrices for each treatment, while a minor mean Ct reduction at T30 minus T0 was observed in the saliva samples versus the NPSs for all the treatments. This latter finding could be because the mean Ct in the saliva samples was already higher at T0 than the mean Ct in the NPSs, and this difference was proportionally maintained in the subsequent observation times. Nonetheless, the average change in Ct between the two matrices remained the same during all follow-up periods. It is worth underlining that the mean Ct values at T30 for both matrices exceeded 40 Ct, indicating a viral load at the limit of detectability.

Overall, the linear regression analyses indicated a high concordance between the Ct values obtained in the saliva and NPS matrices after mAb or antiviral treatment. According to published data, we observed mean Ct values in NPSs lower than in saliva at T0 and at the following time points [30]. However, the mean Ct reduction after drug administration was comparable in both matrices, thus suggesting that saliva could represent a valid alternative matrix for in vivo follow-up studies to evaluate the effectiveness of treatments against COVID-19. Increasing the number of patients, treatments, and follow-up times in studies can help confirm our results and fully define how accurate saliva tests are.

## Data Availability

The original contributions presented in this study are included in the article; further inquiries can be directed to the corresponding author.
